# Effects of early cardiac exercise rehabilitation on the recovery of cardiac function in patients following open heart surgery

**DOI:** 10.3389/fcvm.2026.1906620

**Published:** 2026-10-23

**Authors:** Hong Cai, Lingna Cai, Minhui Ding, Hong Li, Siping Chen

**Affiliations:** 1 Department of Nursing, Fuzhou University Affiliated Provincial Hospital, Fuzhou, Fujian, China; 2 Fuzhou University Affiliated Provincial Hospital, Fuzhou, Fujian, China; 3 Fujian Medical University, Fuzhou, Fujian, China

**Keywords:** cardiac function, cardiac function classification, early cardiac exercise rehabilitation, nursing, open heart surgery

## Abstract

**Introduction:**

This study aims to investigate the short-term effect of early cardiac exercise rehabilitation on the recovery of cardiac function in patients who have undergone open heart surgery.

**Methods:**

A total of 120 patients who performed open heart surgery from January 2019 to December 2021 were divided into an experimental group (*n* = 60) and a control group (*n* = 60). The control group received standard nursing by nurses while the experimental group participated in early cardiac exercise rehabilitation training. The postoperative cardiac function (at discharge and 3 months after operation), gastrointestinal peristalsis recovery, infection rate, readmission rate, and patient satisfaction of both groups were assessed.

**Results:**

The experimental group demonstrated lower infection rate, reduced readmission rate, higher patient satisfaction, and better postoperative cardiac function at 3 months after operation than the control group (*P* < 0.05). There was no significant difference in the postoperative cardiac function at discharge and the recovery time of gastrointestinal peristalsis between two groups (*p* > 0.05).

**Discussion:**

In conclusions, early cardiac exercise rehabilitation training during the perioperative period can help to improve the recovery of cardiac function in a short term for patients who have undergone open heart surgery. At the same time, early cardiac exercise rehabilitation also helped to reduce postoperative infections and readmission, and improve patient satisfaction..

## Introduction

The heart plays a crucial role in maintaining the normal physiological activities. Cardiovascular diseases remain the leading cause of death and disease burden worldwide, especially in the regions of low, low-middle, and middle Socio-demographic Index (1). Open-heart surgery is a primary treatment option for cardiac diseases, and is well recognized for cardiac surgery due to its wide surgical view and definite revascularization effect (2). However, many patients with heart disease often present different degrees of cardiac dysfunction before surgery. In addition, factors such as intraoperative general anesthesia, extracorporeal circulation, non-physiological reperfusion, and cardiac hypothermia may further impact their cardiopulmonary function (2, 3). Therefore, it is essential to find ways to promote cardiac function after open-heart surgery, so as to improve patients' postoperative quality of life.

Early cardiac exercise rehabilitation is a comprehensive intervention to promote a healthy lifestyle, delay or reverse the progression of cardiovascular disease (CVD) (4, 5). Meanwhile, along with health education, strengthening nurse-patient communication, optimizing and improving nursing quality is a kind of nursing that fully reflects humanistic care. Based on the literature review, increasing the intensity of exercise training may help enhance the postoperative recovery of cardiac function (6, 7). In this way, it can help to improve muscle tension, increase coronary blood flow, and improve the quality of life of these patients. However, the absence of professionalism and systematic training in routine rehabilitation nursing training post-surgery, and the patient's poor postoperative acceptance ability after discharge may discontinue the recovery following discharge. During the critical period when patients transition from in-hospital rehabilitation to postoperative rehabilitation outside the hospital after discharge, there is insufficient attention paid to related cardiac rehabilitation training and related reports (8). In addition, a significant care gap exists during the transition from inpatient to outpatient rehabilitation after discharge. Moreover, patients are rarely involved in decision-making and planning during this critical period.

Therefore, this study aims to explore the short-term effect of early cardiac exercise rehabilitation training on the recovery of cardiac function before discharge and 3 months after discharge, with the expectation of providing a reference basis for the clinical construction of safe and continuous rehabilitation care plans for the patients receiving open-heart surgery.

## Methods

### Study design and participants

This study was a quasi-experimental study, which was performed in accordance with the Declarations of Helsinki. This study was approved by the ethics committee of Fujian Medical University (No. K2019-07-014), and written informed consent was obtained from each patient.

The patients who underwent open heart surgery were enrolled continuously in the Cardiac Surgery Department of our hospital from January 2019 to December 2021. The inclusion criteria were: (1) patients diagnosed with heart disease who underwent open-heart surgery, (2) age ≥18 years old, (3) ICU stay ≤48 h, (4) conscious, able to cooperate with rehabilitation treatment, and signed informed consent. Exclusion criteria: (1) patients with mental disorders, (2) critically ill patients requiring reoperation, patients with cerebrovascular disease, severe lung disease or abnormal liver and kidney function; (3) previous history of stroke, chronic lung disease, chronic kidney disease or dialysis, or history of emergency surgery.

### Grouping

Figure 1 shows the flow chart of patients' inclusion. The included patients were divided into the control group (*n* = 60) and the experimental group (*n* = 60) according to their selection of whether to perform early cardiac exercise rehabilitation training. The patients who received standard nursing care post-surgery were included in the control group. In contrast, the patients who adopted early cardiac exercise rehabilitation training in addition to standard nursing care were included in the experimental group.

**Figure 1:**
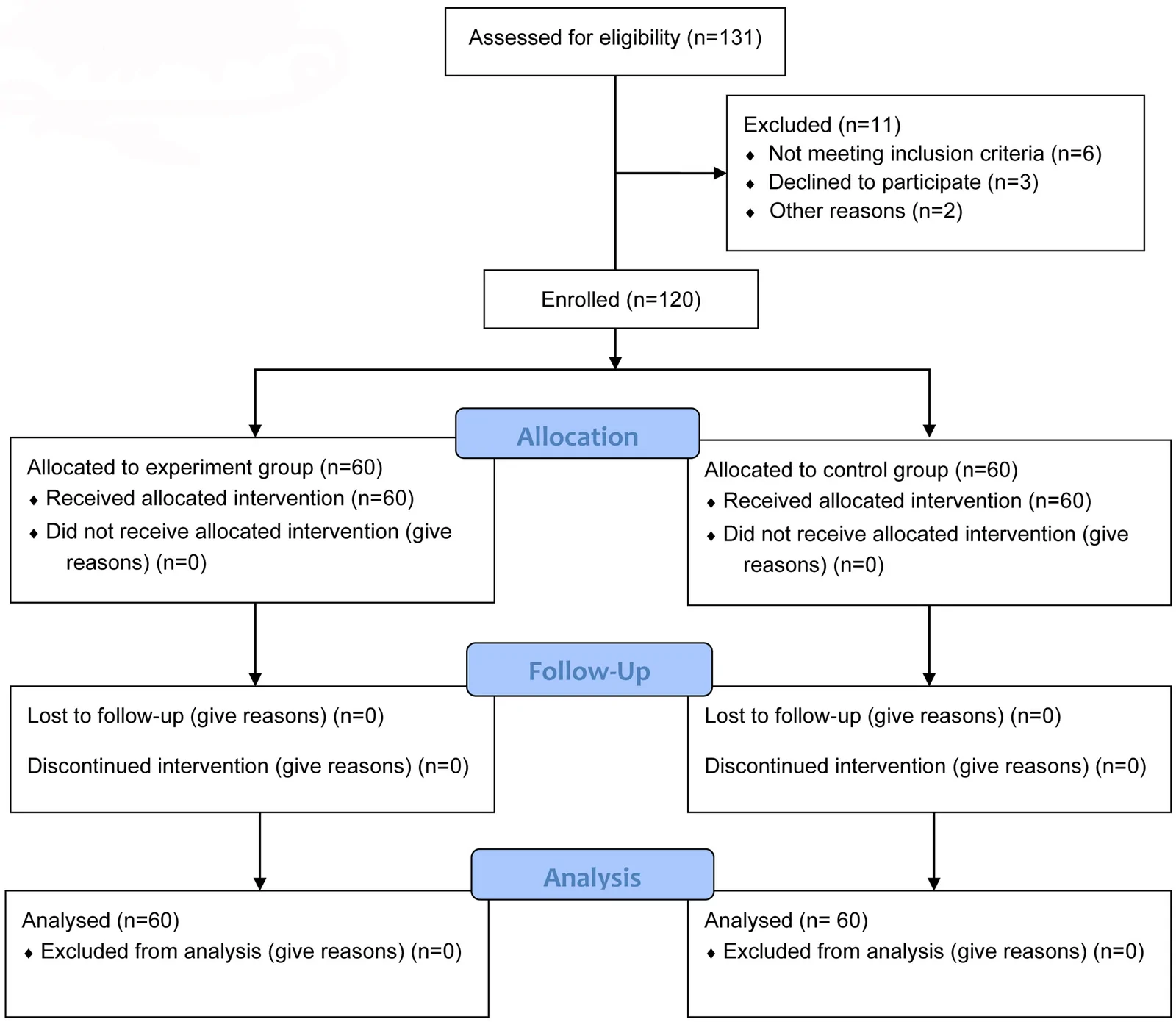
Flow chart of patients' inclusion.

### Interventions

#### Standard nursing care

Standard nursing care post-surgery included the following steps: After the operation, the patient was transferred to the intensive care unit. Close monitoring was carried out for vital signs such as heart rate, blood pressure, breathing, blood oxygen saturation and body temperature. Continuous observation of hemodynamic changes was conducted. The dressing at the incision site of the sternum was kept clean and dry, and any signs of infection were noted. Encouraging the patient to perform early bed activities after the operation was promoted. It was suggested that the author undertake deep breathing exercises to prevent pulmonary complications.

#### Early cardiac exercise rehabilitation training

An early cardiac rehabilitation team was established. The team members include 2 specialized nurses, 1 critical care physician, 1 cardiovascular surgeon, 1 cardiac rehabilitation specialist, and 1 psychological counselor. The early cardiac exercise rehabilitation was a multicomponent early cardiac rehabilitation program, which was proceeded in the inpatient rehabilitation period.

The intervention of early cardiac exercise rehabilitation for the experimental group was developed based on the patient's condition, and was performed according to the following procedures. During the hospitalization period, the cardiac rehabilitation specialist supervised the implementation of the exercises on-site.

The specific interventions on nursing for the first phase were as follows (9): (1) Cardiac rehabilitation nursing evaluation: The data were comprehensively evaluated, and risk factor management included a strict sodium restriction (sodium salt intake <5 g/day), and the control on blood pressure and blood sugar levels. (2) Preoperative respiratory function exercise: Following the instruction of the medical staff, the patient learned to take a deep breath, blow a balloon, and cough effectively, 3–5 times a day for 3–5 days, 5–10 min each time. (3) the cardiac function classification of patients was evaluated (grade I, grade II, grade III, grade IV) for rehabilitation training (10). Early postoperative “four-step” rehabilitation training was developed and performed as follows: The first stage was the 1–2 days after the operation. The rehabilitation training included passive limb exercise, deep breathing exercise and effective expectoration in bed. Patients should perform passive limb exercise 2–3 times a day for 5–10 min each time, with the control of the resting heart rate increased by 10–20 times/min; deep breathing exercise should be performed 3–5 times a day, 10–15 min each time. If the patient has discomfort during this period, including a change in heart rate ≧20 beats/min, a change in blood pressure ≧20 mmHg, or arrhythmia, stop activities immediately and adjust the rehabilitation training plan.

The second intervention stage involved the training activities at the 3rd to 4th days after the operation. Patient should do active movement, active flexion and extension joint movement, lower limb lifting movement, and ankle pump movement on the bed, 10–20 times each time for 2–3 times a day. If the patient has discomfort such as shortness of breath and chest tightness, the training should be discontinued.

The third stage involved getting out of bed, the order of getting out of bed is side lying, getting up, feet on the ground, sitting on the edge of the bed (3 min), standing on the edge of the bed (5 min), and moving away from the bed (10–20 min), walk 50–100 m, 1–2 times a day. These should be performed at 5–7 days postoperatively. If the patient has discomfort such as shortness of breath and chest tightness, the training should be discontinued.

At the fourth stage from the 7th day after the operation to before discharge, the patient gradually completed the self-care activities such as washing, eating, toileting and other self-care activities. Patient also should take some low-intensity aerobic exercises, such as walking 300–500 m for 1–3 times a day, for 20–30 min each time. If the patient has discomfort such as shortness of breath and chest tightness, the training should be discontinued.

The exercise training after discharge was an extension of the inpatient rehabilitation program, and the intensity of the exercise had been adjusted. After discharge, the daily exercise at home was adjusted to 30–60 min. The engaged activities included brisk walking, using elastic bands, stretching, and practicing Tai Chi. This study employed the complete 24-form simplified Tai Chi routine. The participants completed the entire set of Tai Chi, with each training session lasting 20 min per day. Patient was informed to stop the exercise and contact the doctor if he/she felt uncomfortable. The nurses made 2–3 phone calls every week to remind the patients to continue their exercise training at home, but did not record their exercise training. The patients filled out exercise diaries by themselves, but no real-time video supervision was provided during the home-based stage. No other intervention was carried out after discharge. Patients came back to the hospital for double check after 3 months.

### Research tools

(1) General information collection: This included details of sex, age, living conditions, lifestyle, cardiac function classification, type of operation, and duration of the operation. (2) Early Cardiac Rehabilitation Questionnaire: The early cardiac rehabilitation questionnaire was developed based on relevant literature. This questionnaire involves the NYHA cardiac function classification standard, postoperative gastrointestinal peristalsis recovery time (time from postoperative to begin eating), the time taken to get out of bed for the first time post-surgery and postoperative infections. (3) Patient Satisfaction Questionnaire: This questionnaire was self-designed. The total score of the satisfaction questionnaire was 10 points. A score of <4 was deemed as poor satisfaction, a score of 6–8 was moderate, and a score of 10 was good satisfaction.

### Data collection

During hospitalization, observation method was used to collect postoperative gastrointestinal peristalsis recovery time, postoperative first ambulation time, occurrence of infections and cardiac function grading. A questionnaire was used to assess patient's satisfaction prior to discharge. Three months after discharge, cardiac function grading was evaluated on the patient through cardiac color Doppler ultrasound at outpatient reexamination.

### Observation indicators

The main observation indicators for this study included cardiac function classification, and the time to first ambulation post-surgery. The secondary indicators compromised the postoperative recovery time for gastrointestinal peristalsis, infection rate, readmission rate and patient satisfaction.

Postoperative infections included pulmonary infection, and wound infection. Pulmonary infection was determined as newly diagnosed pneumonia that occurred after surgery, confirmed by laboratory examination of inflammatory indicators, chest x-ray examination, and the symptoms of fever, cough or other respiratory symptoms. Postoperative wound infection referred to the infection that occurs in the surgical incision or the tissues, organs or cavities involved in the surgery after the operation. Gastrointestinal peristalsis recovery time referred to the duration required for the intestinal smooth muscle to regain its regular and progressive contraction movement after surgery. Clinically, the recovery was usually marked by the first occurrence of bowel sounds, the first defecation, or the first flatus. First time to get out of bed after operation referred to the patient's earliest postoperative moment independently or with assistance, from lying in bed to sitting at the bedside, standing, or walking. Readmission rate was the proportion of patients who were discharged and subsequently admitted again within 30 days.

### Statistical analysis

Data were processed using SPSS22.0 software. Measurement data are presented as mean ± standard deviation (SD) and assessed using a *t*-test. Count data are expressed as constituent ratios, and a chi-square test was used. Rank data are expressed using the rank sum test; A *P* < 0.05 indicates a statistical significance.

## Results

### General information

There were 36 males and 24 females in the experimental group, aged 27–79 years old with an average age of 57.53 ± 11.31 years old. The experimental group included 34 cases of heart valve surgery, 22 cases of coronary artery bypass surgery, and 4 cases of congenital heart disease. There were 28 males and 32 females in the control group, aged 27–74 years old, with an average age of 57.00 ± 12.58 years. The control group included 39 cases of heart valve surgery, 18 cases of coronary artery bypass surgery, and 3 cases of congenital heart disease surgery. There were 2 cases of combined diabetes and 5 cases of combined hypertension in the experimental group, while there were 3 cases of combined diabetes and 4 cases of combined hypertension in the control group. Both groups of patients showed no statistically significant differences in general information such as age, sex, preoperative cardiac function classification, surgical methods, and combined diabetes and hypertension (*P* > 0.05). All these patients completed the intervention and came back for follow-up visits after 3 months.

### Comparison of the cardiac function classification

The results showed that there was no significant difference in the recovery of cardiac function between the two groups of patients at discharge (*P* = 0.734). However, better cardiac function was observed in the experimental group than the control group at 3 months post operation (*P* = 0.047) (Table 1).

**Table 1:** Comparison of cardiac function recovery before and after discharge.

Groups	Cardiac function before discharge (grade)				Cardiac function at 3 months after operation (grade)			
	Ⅰ	Ⅱ	Ⅲ	Ⅳ	Ⅰ	Ⅱ	Ⅲ	Ⅳ
Experimental group (*n* = 60)	36	18	6	0	44	14	2	0
Control group (*n* = 60)	36	19	5	0	33	19	8	0
Z	0.618				3.927			
P	0.734				0.047			

### Recovery time of gastrointestinal peristalsis

No significant difference was observed in the recovery time of gastrointestinal peristalsis between the two groups after operation (*P* = 0.450), and the experimental group showed a slightly shorter time of the first getting out of bed than the control group (1.73 vs. 1.92 days, *P* = 0.048) (Table 2).

**Table 2:** Comparison of postoperative gastrointestinal peristalsis recovery time and ambulation time.

Groups	Gastrointestinal peristalsis recovery time (days)	First time to get out of bed after operation (days)
Experimental group (*n* = 60)	1.73 ± 1.14	4.83 ± 2.40
Control group (*n* = 60)	1.92 ± 1.40	6.53 ± 3.98
t	0.579	4.093
P	0.450	0.048

### Postoperative infections and readmission

The experimental group exhibited less postoperative infections (*P* = 0.046) and lower postoperative readmission rates (*P* = 0.041) than the control group (Table 3).

**Table 3:** Comparison of infections and readmission rates.

Groups	Postoperative infections (n/%)	Readmission rate (n/%)
Experimental group (*n* = 60)	7 (11.7%)	6 (10.0%)
Control group (*n* = 60)	16 (26.7%)	12 (20.0%)
X^2^	6.004	7.752
P	0.046	0.041

### Patient satisfaction

The patients in the experimental group were better satisfied than those in the control group (*P* = 0.040) (Table 4).

**Table 4:** Comparison of the satisfaction from discharged patients.

Groups	Good satisfaction (n/%)	Moderate satisfaction (n/%)	Poor satisfaction (n/%)
Experimental group (*n* = 60)	36 (60%)	22 (36.7%)	2 (0.03%)
Control group (*n* = 60)	26 (43.3%)	20 (33.3%)	14 (23.3%)
X^2^	−2.054		
P	0.040		

## Discussion

In this study, the experimental group demonstrated improved cardiac function compared to the control group three months after surgery. Additionally, the experimental group experienced fewer postoperative infections and lower readmission rates. Furthermore, patients in the experimental group reported higher satisfaction levels than those in the control group.

Open-heart surgery may lead to a high risk of postoperative complications and a long recovery period, which results in physical and mental stress for patients (1–3). Early cardiac rehabilitation can improve patient outcomes and speed up recovery (4, 5). Open-heart surgery is performed in the context of cardiopulmonary bypass. During cardiopulmonary bypass, cardiac blood flow is temporarily interrupted, and some myocardial cells are severely damaged, resulting in restrict cardiac function. Early goal-directed exercises could be related to enhanced balance, functionality, and high physical health-related quality of life among patients with cardiac surgery (11, 12). Chai et al. (13) find that 36.8% of the post-PCI patients lack of exercise in the adherence study of phase I cardiac rehabilitation. Early exercise rehabilitation training can enhance cardiac function recovery (4, 6). This study showed that the recovery of cardiac function was better among the patients in the experimental group 3 months after the operation than the control group, along with less infections and lower readmission rate after discharge. These results were consistent with the previous reports in cardiovascular surgeries (4, 6). A recent meta-analysis involving 25 randomized controlled trials with 4,106 participants finds that, cardiac rehabilitation based on exercise significantly reduces hospitalization rates and the incidence of myocardial infarction, while improving health-related quality of life (14). This further supports our research findings.

Open-heart surgery can significantly affect the patient's mental state, triggering a stress response in the body, especially in the gastrointestinal (15–17). There was no statistical significance in the recovery time of gastrointestinal peristalsis between the two groups in this study. Rare literature reports about gastrointestinal recovery after intervention nursing in patients who undergo open-heart surgery. The similar recovery time in this study may be related to the small sample size and incomplete analysis of related risk factors, which needs further research.

Early cardiac exercise rehabilitation training helps patients get out of bed faster. It includes postural training, limb activities, and aerobic exercise. These exercises improve breathing, maintain joint movement, prevent muscle atrophy, and increase exercise capacity and cardiopulmonary function (7, 18). Ceylan et al. (11) reports that early mobilization and functional exercise significantly enhance balance, functionality, and quality of life in a randomized controlled trial involving elderly cardiac surgical patients. In this study, the first time to get out of bed after surgery is earlier in the experimental group than the control group, indicating that early cardiac exercise rehabilitation training can improve clinical status.

Early cardiac exercise rehabilitation training reduces postoperative complications in patients. After open heart surgery, patients are at risk for complications like pulmonary infection. Training helps patients heal and may improve respiratory function by increasing airway pressure and reducing infection risk (19). In this study, lower incidence of postoperative pulmonary infection was observed in the experimental group than the control group, indicating the effectiveness of the rehabilitation exercise. Early respiratory function training could reduce the incidence of infections to a certain extent. A systematic review of exercise-based prehabilitation in patients undergoing coronary artery bypass grafting shows that structured exercise programs effectively reduce postoperative complications, further supporting our findings (20). At the same time, smoking cessation guidance and cardiovascular knowledge were given and related rehabilitation knowledge education can improve patient compliance and satisfaction, but there is no specific standard for specific lung function exercise frequency.

This study has several limitations. First, it was conducted at a single center study with only a 3-month follow up. Second, this study did not account for the confounding factors and other risk factors that may impact the patients' cardiac function. Third, this study did not intervene with the nutrition, training plan and lifestyle of the patients during the period after discharge, which may introduce some bias to the results. Fourth, this study relies largely on qualitative measures, which may affect the robustness and reliability of the findings. Fifth, the lack of objective exercise-adherence monitoring, and the multicomponent nature of the intervention may lead to some bias in the results. Therefore, a multi-center study with a long-term follow up, incorporating an analysis of confounding factors, nutrition and lifestyle, is necessary to validate the effectiveness of the early cardiac rehabilitation plan.

## Conclusion

In summary, studies have shown that early cardiac rehabilitation can promote the recovery of cardiac function, shorten the time to get out of bed for the first time, and significantly reduce the incidence of postoperative infections and readmission rates. Due to factors such as single-center trial, short-term follow up, qualitative measures, and limited interventions after discharge, these findings require further investigations to validate.

## Data Availability

The original contributions presented in the study are included in the article/Supplementary Material, further inquiries can be directed to the corresponding author.
